# Brief isoflurane administration as an adjunct treatment to control organophosphate-induced convulsions and neuropathology

**DOI:** 10.3389/fphar.2023.1293280

**Published:** 2023-12-05

**Authors:** Narayanan Puthillathu, John R. Moffett, Alexandru Korotcov, Asamoah Bosomtwi, Ranjini Vengilote, Jishnu K. S. Krishnan, Erik A. Johnson, Peethambaran Arun, Aryan M. Namboodiri

**Affiliations:** ^1^ Department of Anatomy, Physiology, and Genetics, Neuroscience Program and Molecular and Cell Biology Program, Uniformed Services University of the Health Sciences, Bethesda, MD, United States; ^2^ Department of Radiology and Radiological Sciences, Uniformed Services University of the Health Sciences, Bethesda, MD, United States; ^3^ The Henry M. Jackson Foundation for the Advancement of Military Medicine (HJF), Bethesda, MD, United States; ^4^ Department of Neuroscience, United States Army Medical Research Institute of Chemical Defense, Gunpowder, MD, United States

**Keywords:** paraoxon, magnetic resonance imaging, convulsant antidote for nerve agents, mean diffusivity, brain edema, acetylcholinesterase

## Abstract

Organophosphate-based chemical agents (OP), including nerve agents and certain pesticides such as paraoxon, are potent acetylcholinesterase inhibitors that cause severe convulsions and seizures, leading to permanent central nervous system (CNS) damage if not treated promptly. The current treatment regimen for OP poisoning is intramuscular injection of atropine sulfate with an oxime such as pralidoxime (2-PAM) to mitigate cholinergic over-activation of the somatic musculature and autonomic nervous system. This treatment does not provide protection against CNS cholinergic overactivation and therefore convulsions require additional medication. Benzodiazepines are the currently accepted treatment for OP-induced convulsions, but the convulsions become refractory to these GABA_A_ agonists and repeated dosing has diminishing effectiveness. As such, adjunct anticonvulsant treatments are needed to provide improved protection against recurrent and prolonged convulsions and the associated excitotoxic CNS damage that results from them. Previously we have shown that brief, 4-min administration of 3%–5% isoflurane in 100% oxygen has profound anticonvulsant and CNS protective effects when administered 30 min after a lethal dose of paraoxon. In this report we provide an extended time course of the effectiveness of 5% isoflurane delivered for 5 min, ranging from 60 to 180 min after a lethal dose of paraoxon in rats. We observed substantial effectiveness in preventing neuronal loss as shown by Fluoro-Jade B staining when isoflurane was administered 1 h after paraoxon, with diminishing effectiveness at 90, 120 and 180 min. *In vivo* magnetic resonance imaging (MRI) derived T2 and mean diffusivity (MD) values showed that 5-min isoflurane administration at a concentration of 5% prevents brain edema and tissue damage when administered 1 h after a lethal dose of paraoxon. We also observed reduced astrogliosis as shown by GFAP immunohistochemistry. Studies with continuous EEG monitoring are ongoing to demonstrate effectiveness in animal models of soman poisoning.

## 1 Introduction

OP-based compounds are highly toxic acetylcholinesterase inhibitors that cause excitotoxic acetylcholine-mediated neuronal excitation, status epilepticus, severe neuropathology (McLeod et al., 1984), behavioral impairment (Yanagisawa et al., 2006) and can rapidly lead to death if left untreated. Current standard treatments can be effective if given rapidly and aggressively. Intramuscular injections of atropine sulfate and 2-PAM limit cholinergic overactivation in the periphery, thus helping to prevent mortality, but due to low blood brain barrier permeability they do not prevent convulsions, seizures and the attendant neuronal damage. Additional treatment with benzodiazepines, including diazepam and midazolam, are currently used to control OP-induced convulsions, though efficacy rapidly declines until becoming refractory approximately 40 min after convulsion onset (McDonough et al., 2010; Kuruba et al., 2018). At this point, other excitatory neurotransmitter systems including glutamate become dominant and propagate seizures (McDonough and Shih, 1997), while at the same time activation of inhibitory neurotransmitter systems, such as GABA, are exhausted and insufficient to reduce the increased excitotoxic signals and therefore, seizures subsequently return (Apland et al., 2014). Repeated use of benzodiazepines has diminishing effects in controlling OP-induced seizures and convulsions as GABA_A_ receptors are saturated and desensitized, leading to insufficient inhibitory feedback to control the ever-increasing excitotoxic signals. It has also been shown that benzodiazepines are less effective at controlling OP-induced seizures than glutamate receptor antagonists (Apland et al., 2014). Failure to completely control seizures leads to long term behavioral deficits in severely affected patients. Prolonged, uncontrolled seizures and convulsions cause severe brain damage, permanent cognitive impairment and can lead to death. The small therapeutic window for benzodiazepines highlights the need for new treatments that can control seizures and convulsions through mechanisms distinct from the GABA_A_ receptor. Adjunct therapies that can offer improved anticonvulsant efficacy with a longer therapeutic window would improve survival rates and protect long-term neurological function.

One potential therapeutic is isoflurane, a volatile halogenated ether that is a common anesthetic in clinical use. While isoflurane acts as a positive allosteric modulator of the GABA_A_ receptor, it also potentiates inhibitory glycine receptor activity and inhibits excitatory NMDA glutamate receptor activity (Nishikawa and MacIver, 2000; Grasshoff and Antkowiak, 2006). Isoflurane also acts through TASK-3 potassium channels to repolarize neuronal membranes (Luethy et al., 2017) which may play some role in isoflurane’s neuroprotective effects. We have shown that very brief (4 min) high dose (3%–5%) isoflurane has potent anticonvulsant and neuroprotective efficacy in a paraoxon model of OP poisoning (Krishnan et al., 2017). This short-duration, high-dose application of isoflurane is opposite of its use as an anesthetic, where low doses are given for extended periods to maintain surgical anesthesia. Neither diazepam nor midazolam reduce mortality in rats administered a lethal dose of paraoxon (Krutak-Krol and Domino, 1985), whereas we found that isoflurane eliminated all mortality (37% untreated, 0% isoflurane treated) in the same animal model (Krishnan et al., 2017). Recently, the repurposing of isoflurane for use in controlling seizures in epilepsy patients has been proposed (Klein et al., 2020). However, to date, only our research group has proposed the use of brief, high-dose isoflurane as a potent and effective anticonvulsant for OP exposure. This brief isoflurane treatment rapidly stops convulsions and protects vulnerable brain regions from excitotoxic damage, without the need to be re-administered as is the case with benzodiazepines. The greater efficacy of isoflurane in blocking OP-induced seizures and convulsions is that it works through multiple mechanisms simultaneously, rather than acting only on the GABA_A_ receptor system (Ranft et al., 2004; Neag et al., 2020; Pavel et al., 2020). Here we use *in vivo* MRI to demonstrate the neuroprotective effects of isoflurane when administered 1 h after a lethal dose of paraoxon, as well as providing an extended time course up to 180 min after paraoxon in reducing neuronal damage using Fluoro-Jade B (FJB) staining 24 h after injury. We also show that astrogliosis resulting from paraoxon poisoning is reduced by brief isoflurane administration.

## 2 Materials and methods

Animal experiments were conducted following guidelines from the NIH for the care and use of laboratory animals, and the protocols were approved by the animal care and use committee of the Uniformed Services University of the Health Sciences, Bethesda, MD. Seven-week-old adult male Sprague-Dawley rats, (250 ± 40 g), were used for all studies (Taconic Biosciences, NY). Animals were housed in individual cages in environmentally controlled rooms (20–23^o^C, ∼44% humidity, 12 h light/dark cycle, 350–400 lx, lights on at 6:00 a.m.), with food (Teklad Global; 18% protein #2018 rodent diet; Harlan Laboratories, IN) and water available continuously. Animal handling was minimized to reduce stress. Reagents were from Sigma Aldrich (St. Louis, MO).

### 2.1 Paraoxon administration

Paraoxon solutions were prepared fresh by adding 10 µL of stock solution (paraoxon ethyl; 1.27 g/mL) to 3 mL of ice-cold phosphate buffered saline and mixing thoroughly. Paraoxon solutions were kept on wet ice until used. A lethal dose of paraoxon (4 mg/kg; approximately 10x the LD_50_ in rats) (Deshpande et al., 2014; Misik et al., 2015) was administered subcutaneously, followed immediately (within 30 s) by intramuscular atropine sulfate (2 mg/kg) and intramuscular 2-PAM (25 mg/kg).

### 2.2 Racine scale convulsion monitoring

Rats were observed independently by two trained researchers for signs of convulsion onset. The animals were monitored continuously for convulsion severity according to a modified Racine Scale: Stage 0, no behavioral response; Stage 1, behavioral arrest, orofacial movements, chewing; Stage 2, head nodding/myoclonus; Stage 3, unilateral/bilateral forelimb clonus without rearing, straub tail, extended body posture; Stage 4, bilateral forelimb clonus plus rearing; Stage 5, rearing and falling; Stage 6, full tonic seizures (Dorandeu et al., 2013; Krishnan et al., 2017). Results for each animal were recorded at multiple time points ranging from 30 min to 8 h after paraoxon administration.

### 2.3 Administration of isoflurane

Our approach to treat OP poisoning exploits the powerful effects of isoflurane in counteracting OP-induced convulsions when administered for short durations at high doses. The doses used to initially induce anesthesia with isoflurane are substantially higher than the doses used to maintain anesthesia. In rats, the maximal dose used to induce anesthesia for isoflurane is 5% delivered in 100% oxygen in an anesthesia chamber (Miller et al., 2016) and this is the dose we used in most of this study. For dose-response studies we used behavioral testing (Racine Scale) to determine if lower doses of isoflurane (4% and 4.5%) had the same anticonvulsant properties. In the MRI experiments we used 5% isoflurane in 100% oxygen for 5 min, delivered 1 h after paraoxon. For the neuropathology time course studies, we administered 5% isoflurane in 100% oxygen for 5 min at several time points after paraoxon (60, 90, 120, and 180 min). Control animals were not treated with 2-PAM, atropine sulphate or 100% oxygen.

### 2.4 MRI

MRI experiments were conducted 24 h after paraoxon exposure and evaluated for brain hyper-intense parenchymal lesions and parenchymal edema using a 7T Bruker Biospec 70/20 (Bruker Biospin, Billerica, MA) equipped with a 86 mm quadrature transmit coil and a dedicated 4-element phased array coil. For MRI experiments, 17 animals were given a lethal dose of paraoxon (4 mg/kg) immediately followed by atropine sulfate and 2-PAM. Additional groups included 7 animals acting as uninjured controls (no treatment) and another 7 animals given paraoxon immediately followed by atropine sulfate and 2-PAM and then 60 min later they were administered 5% isoflurane in 100% oxygen for 5 min. Twenty-four hours after paraoxon administration, animals were anesthetized with a mixture of isoflurane/medical air (3% for induction and 1%–2% for maintenance) via a nose cone. The animals were placed in a prone position on a dedicated rat bed with a circulating warm-water circuit to maintain body temperature. Respiration rate and rectal temperature were continuously monitored through the experiments (SA-instruments, Stony Brook, NY). The whole-brain 2D multi-echo Rapid Acquisition with Relaxation Enhancement (2D RARE) sequence with repetition time TR = 5,500 ms, effective echo time TE = 10, 30, 50, 70, 90, 110 ms, rare factor 2, in-plane resolution 150 × 150 µm^2^, 38 slices, slice thickness Thk = 750 μm, was used for T2 map computation to assess edema. Diffusion tensor imaging was used to compute MD based on 2D single-shot echo planar imaging acquisition, TR = 8,000 ms, TE = 32 ms, 15 diffusion directions, b = 1,000 s/mm2, Δ/δ = 12/4 ms, in-plane resolution 300 × 300 µm^2^, 38 slices, Thk = 750 µm.

### 2.5 Data processing and statistics

All 3D T2W images were converted to NIFTI format using BrkRaw python module (DOI: 10.5281/zenodo.6803744). A bias field correction was performed on the T2W MR images using the N4BiasFieldCorrection from Advanced Normalization Tools (ANTs) package (Avants et al., 2010). The preliminary brain masks were calculated using atlasBREX (Lohmeier et al., 2019) on the T2W (TE = 30 ms) images, followed by careful quality control of brain segmentation with minor adjustments in areas where the algorithm was unable to clearly separate brain tissue from the rest of the head. Estimates of T2-decay ( $T_{2}$ ) and amplitude ( $S_{0}$ ) images were obtained from 2D multi-echo multi-slice T2W RARE images using a nonlinear fitting algorithm, written in Matlab (Mathworks R2019b, Nattick, MA), from the equation $S_{i}=S_{0}e^{-\frac{TE_{i}}{T_{2}}}$ , where $S_{i}$ is the signal intensity for echo time $TE_{i}$ .

Diffusion tensor images were processed using the TORTOISE v3.2.0 software package (https://tortoise.nibib.nih.gov). Imaging data was analyzed using an atlas-based approach. A rat template from VivoQuant (https://www.vivoquant.com/) with corresponding atlas was automatically registered to T2W skull-stripped image (TE = 30 ms) for each subject using an affine transformation and the nonlinear registration algorithm in ANTs (Avants et al., 2010). Average values were computed within each atlas region. The T2 and MD maps in neocortex region of the atlas were analyzed. All statistical analyses were performed using GraphPad Prism (GraphPad Software, Boston, MA). Two-way ANOVA statistical tests (Šídák’s multiple comparisons with significance determined at *p* < 0.05) were used for analysis.

### 2.6 Time course of isoflurane effectiveness in preventing neuropathology

To assess the effectiveness of isoflurane at different time points, we examined FJB staining twenty-four hours after paraoxon administration. Experimental groups included uninjured controls (n = 3), paraoxon without isoflurane (n = 4) and rats administered 5% isoflurane at several post-paraoxon time points including 60 min (n = 3), 90 min (n = 3), 120 min (n = 3) and 180 min (n = 3). Twenty-4 hours later, rats were deeply anesthetized with a pentobarbital-based preparation (100 mg/kg, i.p.) and transcardially perfused with 4% freshly depolymerized paraformaldehyde (200–300 mL each, pH 7.4). Brains were extracted carefully and post-fixed overnight in the same fixative before being transferred to 30% sucrose for cryoprotection. Brains were sectioned by cryostat at a thickness of 30 μm, and 11 to 12 tissue sections were collected in the region from −2.0 to −2.8 mm from Bregma. Neuronal loss was visualized in FJB-stained sections as described previously (Krishnan et al., 2016; Krishnan et al., 2017). A complete digital representation of each tissue section was acquired with a 10x objective using a Zeiss Axioscan Z1 automated slide scanner. Neuronal injury was scored for all imaged tissue sections using a 10-point scale (1 = minimal injury, 10 = maximal injury) by visual inspection. For each animal, all 11–12 tissue sections were examined and each of the areas of interest was scored according to the degree of FJB staining. If any FJB stained neurons were observed in the area, a score of 1 was assigned. A score of 10 was assigned based on the most extensive FJB staining observed in that region among all animals, with intermediate scores assigned in approximate 10% increments. Statistical analysis was done using SigmaPlot 12.5.

### 2.7 Immunohistochemistry for astrogliosis

Additional rats (n = 3 per group) were processed for glial fibrillary acidic protein (GFAP) immunohistochemistry to monitor astrogliosis after paraoxon poisoning. Isoflurane (5% for 5 min in 100% oxygen) was administered 1 h after paraoxon, and 24 h later the animals were deeply anesthetized with a pentobarbital-based preparation (100 mg/kg, i.p.) and perfused as noted above for FJB staining. GFAP immunohistochemistry was done as previously described (Appu et al., 2017).

## 3 Results

### 3.1 Racine scale behavioral monitoring

**FIGURE 1:**
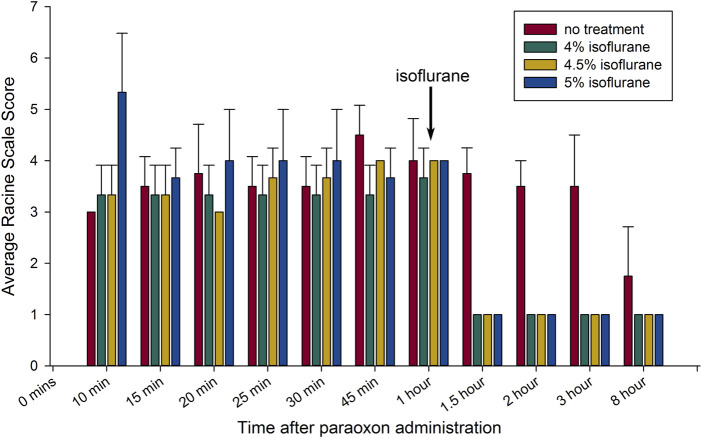
Brief isoflurane administration rapidly halts convulsions up to 8 h: The anticonvulsant response to isoflurane when administered 1 h after paraoxon exposure are shown. The bar graph shows means ± SEM for seizure severity based on the Racine scale used (n = 3 for each group). Each group is represented by a bar and average seizure severity scored at each time of observation. The arrow indicates the time of initiation of isoflurane administration. All groups except untreated controls had brief 5 min isoflurane administration (in 100% oxygen) at the indicated concentrations, initiated 1 h after paraoxon (all treated groups after isoflurane administration; *p* < 0.05 in comparison to the control; Mann-Whitney test).

Dose-response experiments were done in rats subjected to paraoxon poisoning. Delivering isoflurane at 5% in 100% oxygen can depress respiration if continued for 15 min or longer, but we did not observe any respiratory depression during the brief, 5-min exposure. Rats were treated 1 h after paraoxon administration with 4%, 4.5% or 5% isoflurane in 100% oxygen for 5 min. Paraoxon injury groups were treated with 2-PAM and atropine sulfate, but were not treated with isoflurane. Animals treated for 5 min with isoflurane at the 1-h post-exposure time point regained consciousness within several minutes after cessation of isoflurane and only exhibited stage 1 convulsive activity afterwards, typically chewing motions (Figure 1). These animals remained awake but mostly motionless for approximately 45–60 min after isoflurane treatment and then displayed low activity afterwards. Unlike the isoflurane treated groups, the untreated group continued variable convulsive activity for hours (Figure 1). After 8 h, the surviving untreated rats continued to show mild convulsive activity and were extremely lethargic. The animals in the isoflurane treatment groups appeared normal at this time point. We did not observe any differences in the anticonvulsant effectiveness among the 3 doses of isoflurane used. These results indicate that the lower dose of 4% isoflurane is as effective at halting convulsions as the higher doses.

### 3.2 T2 values measurements

**FIGURE 2:**
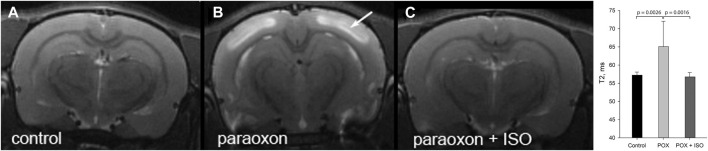
T2 weighted MRI imaging in live rats: T2W images showing extensive cortical edema 24 h after paraoxon (e.g., arrow in **(B)**, and 100% protection when isoflurane (5% for 5 min) was administered 1 h after paraoxon **(C)**. T2W image from a control animal is shown in **(A)**. Long duration exposure was not necessary for neuroprotection: administration for 5 min was highly effective. The maximal safe isoflurane dose (5%), delivered in 100% oxygen, was found to be the most effective at halting convulsions and preventing neurodegeneration. This administration regimen was effective up to 1 h after paraoxon administration (representative images from n = 7 control, 17 paraoxon and 7 paraoxon + isoflurane). Graph inset: averaged T2 values in neocortex at 24 h after paraoxon administration. Significant T2 hyperintensity and T2 values increase were observed in the group given paraoxon, but was not seen in the control or isoflurane treated groups (ISO = isoflurane, POX = paraoxon). Error bars indicate standard deviation.

We examined brain edema 24 h after severe paraoxon poisoning using MRI. Edema presents as high intensity signals in T2W MRI scans as seen in Figure 2B (e.g., arrow on neocortex). Edema was also observed in other brain regions including the hippocampus and piriform cortex. Administration of 5% isoflurane for 5 min, given 1 h after paraoxon, prevented edema (Figure 2C). Comparative T2 values for neocortex are given in Figure 2 (graph inset) for the three experimental groups. T2 values were significantly elevated in neocortex in the group given paraoxon, but not treated with isoflurane. In contrast, in the group given isoflurane (5% for 5 min) 1 hour after paraoxon, the T2 values were comparable to the control group not given paraoxon.

### 3.3 Mean Diffusivity

**FIGURE 3:**
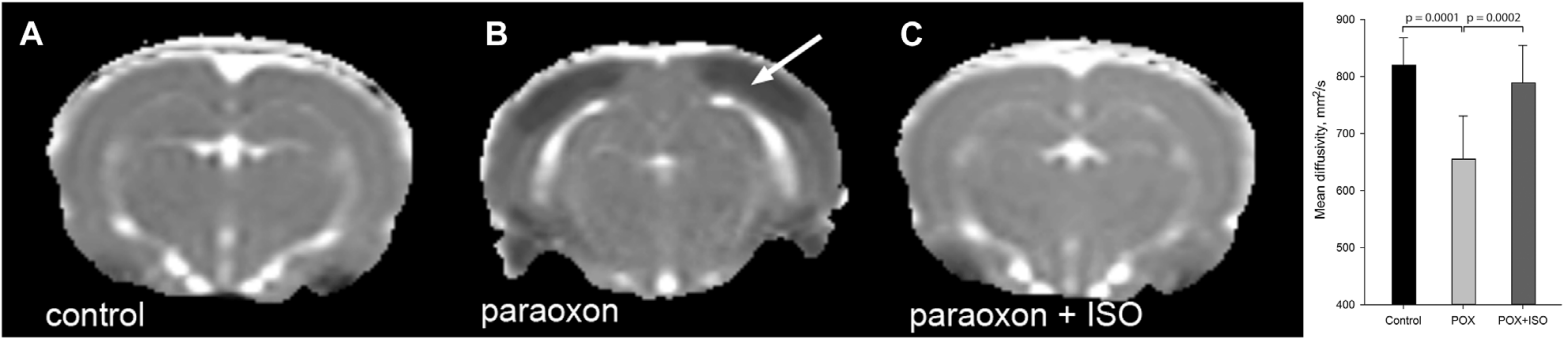
Representative mean diffusivity (MD) maps from the 3 groups 24 h after paraoxon administration: MD measurements showed significant reductions in regions such as neocortex (arrow) in the group given paraoxon, but not treated with isoflurane **(B)**. MD values were similar in control **(A)** and paraoxon + isoflurane **(C)** groups (representative images from n = 7 control, 17 paraoxon and 7 paraoxon + isoflurane). Mean Diffusivity values in neocortex 24 h after paraoxon are shown in the inset graph. MD values were significantly lower in the paraoxon group (POX) than in the control or paraoxon + isoflurane group (POX + ISO). There was no significant difference between the control and POX + ISO groups. Error bars indicate standard deviation.

Mean diffusivity (MD) measurements in the three experimental groups showed significantly decreased cortical MD in the paraoxon group that was not treated with isoflurane (Figure 3). MD reductions were noted in the neocortex, and were also observed in other brain regions, including amygdala, piriform cortex and hippocampus. Reductions in MD values correlate with acute neuronal death and are therefore useful for assessing neuron loss after brain injury (Lee et al., 2020) and define the underlying edema type (Keep et al., 2017). Average MD values in neocortex are given in Figure 3 (graph inset).

### 3.4 Neuropathology

**FIGURE 4:**
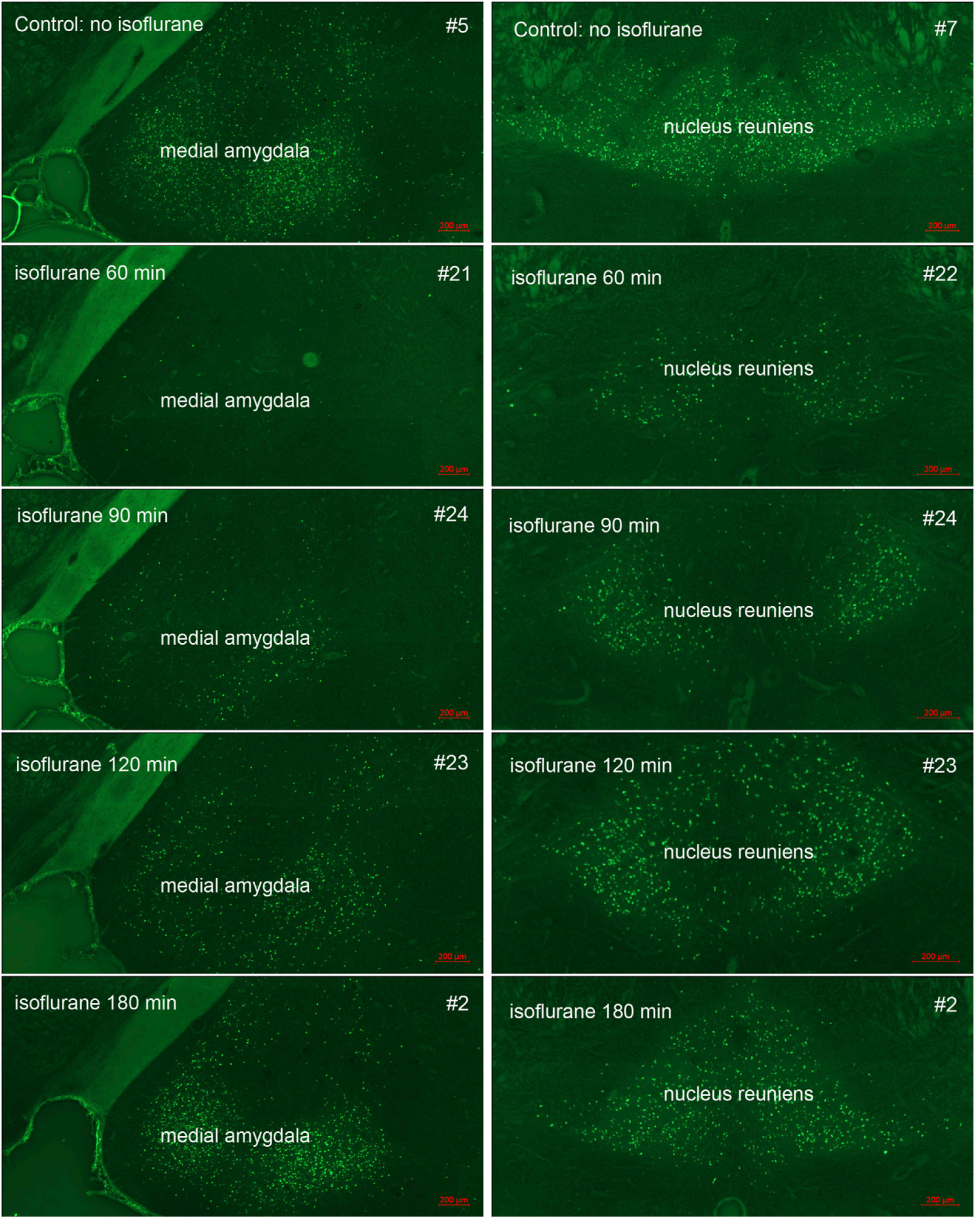
Post-exposure time-course of isoflurane administration in the amygdala and nucleus reuniens: Rats were given a lethal dose of paraoxon (4 mg/kg) and then were administered atropine sulfate and 2-PAM. Isoflurane (5% with 100% oxygen) was administered once for 5 min at 60, 90, 120 or 180 min after paraoxon administration. FJB-stained sections show the neuroprotective effect of isoflurane at each post-exposure time point. Neuronal loss was low to absent in the amygdala, and was reduced in the nucleus reuniens at the 60 min time point. Increasing neurodegeneration was observed at the later time points. Representative images from n = 3 per group, except for the group that was not given isoflurane, where n = 4 (animal tag numbers are given for each image; compare with Figure 5).

**FIGURE 5:**
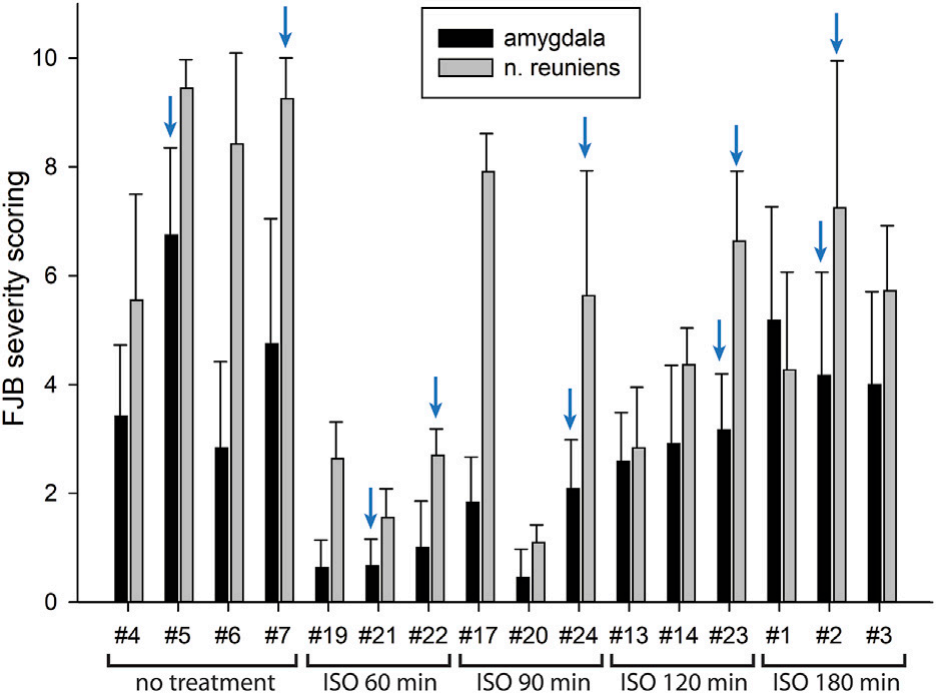
FJB severity scoring by animal in the amygdala and nucleus reuniens. Each bar represents the scoring results from the two locations in each experimental animal. Error bars represent the standard deviation in severity score among 9–12 tissue sections from each animal. Animal tag numbers and treatment groups are shown on the X-axis. ISO = isoflurane. The blue arrows indicate which animals the FJB images in Figure 4 were taken from.

**FIGURE 6:**
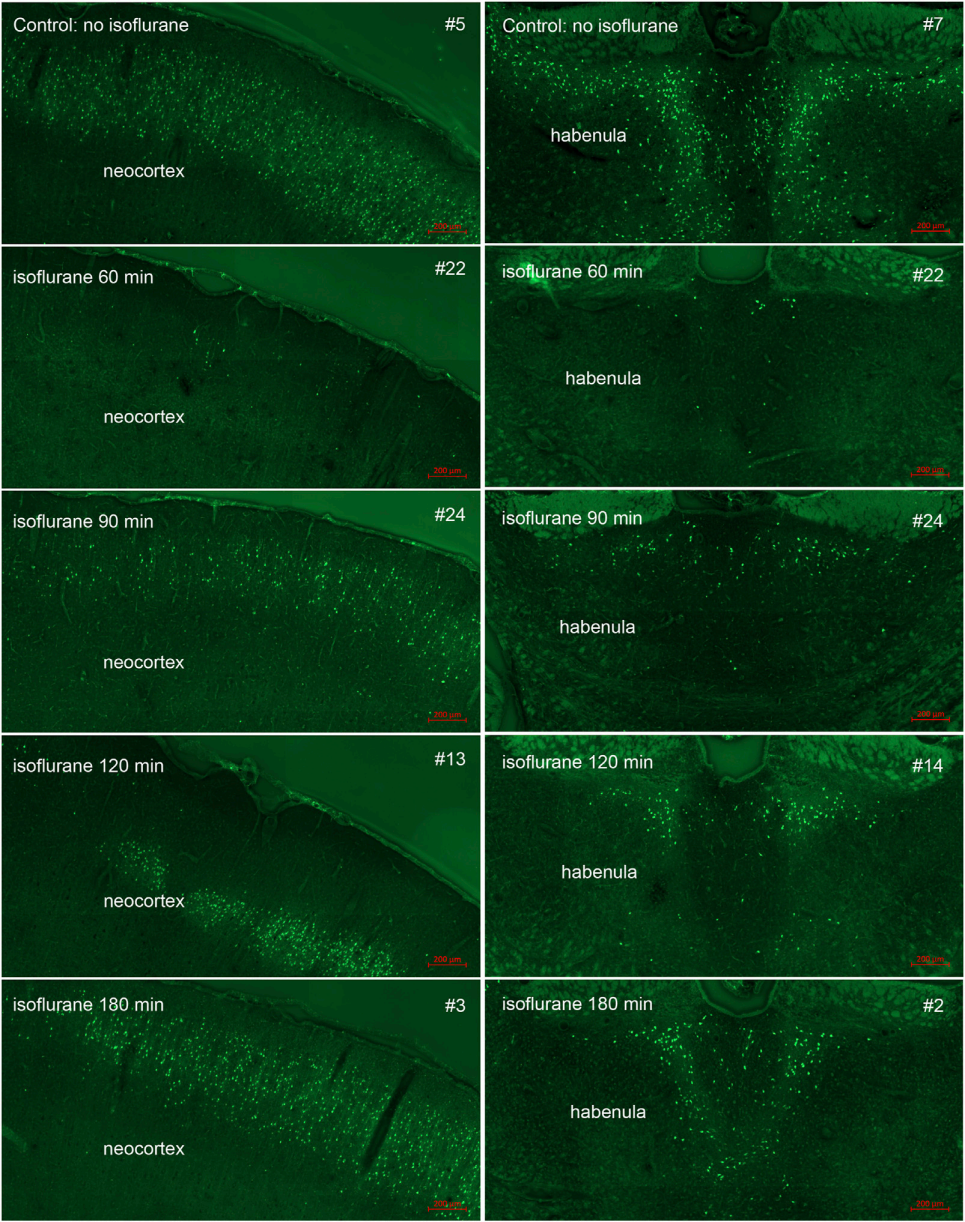
Post-exposure time-course of isoflurane administration in neocortex and dorsal thalamus (habenula): Rats were given a lethal dose of paraoxon (4 mg/kg) and then were administered atropine sulfate and 2-PAM. Isoflurane (5% with 100% oxygen) was administered once for 5 min at 60, 90, 120 or 180 min after paraoxon administration. FJB-stained sections show the neuroprotective effect of isoflurane at each post-exposure time point. Neuronal loss was minimal at the 60 min time point. Increasing neurodegeneration was observed at the later time points. Representative images from n = 3 per group, except for the group that was not given isoflurane, where n = 4 (animal tag numbers are given for each image; compare with Figure 7).

**FIGURE 7:**
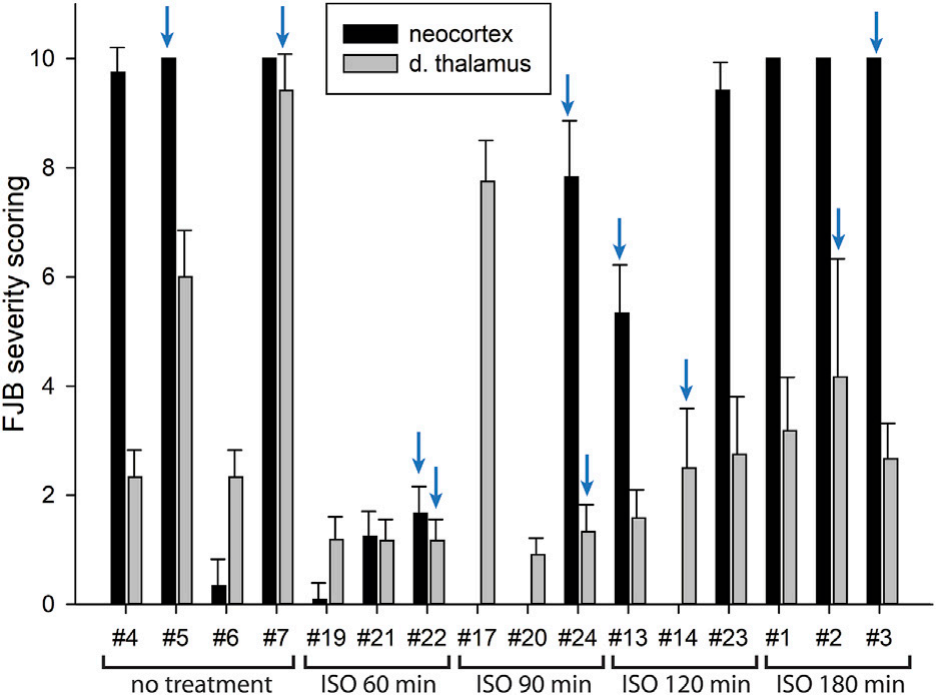
FJB severity scoring by animal in neocortex and dorsal thalamus. Each bar represents the scoring results from the two locations in each experimental animal. Error bars represent the standard deviation in severity score among 9–12 tissue sections from each animal. Animal tag numbers and treatment groups are shown on the X-axis. Note that animals #17, #20 and #14 had no detectable neocortical staining. Two of the three animals in the group given isoflurane at 90 min had no detectable FJB staining in neocortex, whereas the image shown in Figure 6 is from the one animal that had substantial FJB staining in neocortex (animal #24). ISO = isoflurane. The blue arrows indicate which animals the FJB images in Figure 6 were taken from.

**FIGURE 8:**
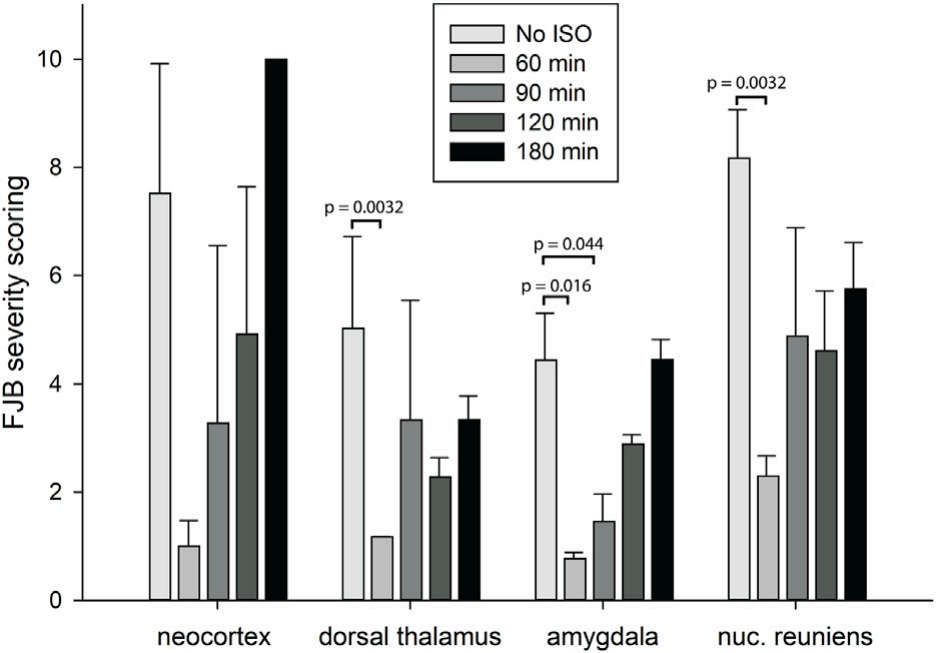
Analysis of the FJB severity scoring by brain region. FJB severity scores were analyzed to determine statistically significant differences in FJB severity scoring in 4 different brain regions at 4 different isoflurane administration time points. Bars represent FJB staining severity scores in 9–12 tissue sections for each animal and 3-4 animals per group (error bars represent standard error). Significantly different FJB scores are shown (*one way ANOVA on ranks, *p* < 0.05).

In our previous studies we showed that 3% isoflurane administered in 100% oxygen prevented all neuronal loss as shown by FJB staining when given 30 min after a lethal dose of paraoxon (Krishnan et al., 2017). Here we examine an extended post-exposure time course of isoflurane’s effectiveness in preventing acute neuronal loss after lethal paraoxon administration. Control animals that did not receive paraoxon or isoflurane had no detectable FJB staining in neurons in any brain region. In animals given paraoxon but not treated with isoflurane, extensive FJB staining was observed in many regions of the brain. We focused on four brain regions that most reliably exhibited extensive FJB staining in our paraoxon model. These included 1) the amygdala complex region lateral to the optic tract, 2) the nucleus reuniens in the central thalamus, just above the 3^rd^ ventricle, 3) the dorsal thalamus below the stria medullaris, including the habenula, and 4) parietal neocortex. We found that 5% isoflurane delivered for 5 min was very neuroprotective when administered 1 h after paraoxon. Neuronal loss, as shown by FJB staining at the 24-h time point, was greatly reduced or eliminated throughout the brain at this isoflurane administration time point. Neuronal staining was negligible in the amygdala and was reduced substantially in the central thalamus (nucleus reuniens) when isoflurane was administered at 1 h after paraoxon (Figures 4, 5). In neocortex and the dorsal thalamus (including habenula), FJB staining was very low at the 60-min administration time point (Figures 6, 7). Partial neuroprotection was observed when isoflurane was administered 90 min after paraoxon, with diminishing effectiveness at the 120- and 180-min time points. FJB staining was visually scored in the four brain regions, and while there was substantial variability in the degree of FJB staining between animals, the trend of neuroprotection was apparent (Figures 5, 7). FJB severity scoring indicated that the neuropathology was significantly reduced in the 60-, 90- and 120-min time points in all 4 regions, relative to the untreated group (Figure 8).

### 3.5 Astrogliosis

**FIGURE 9:**
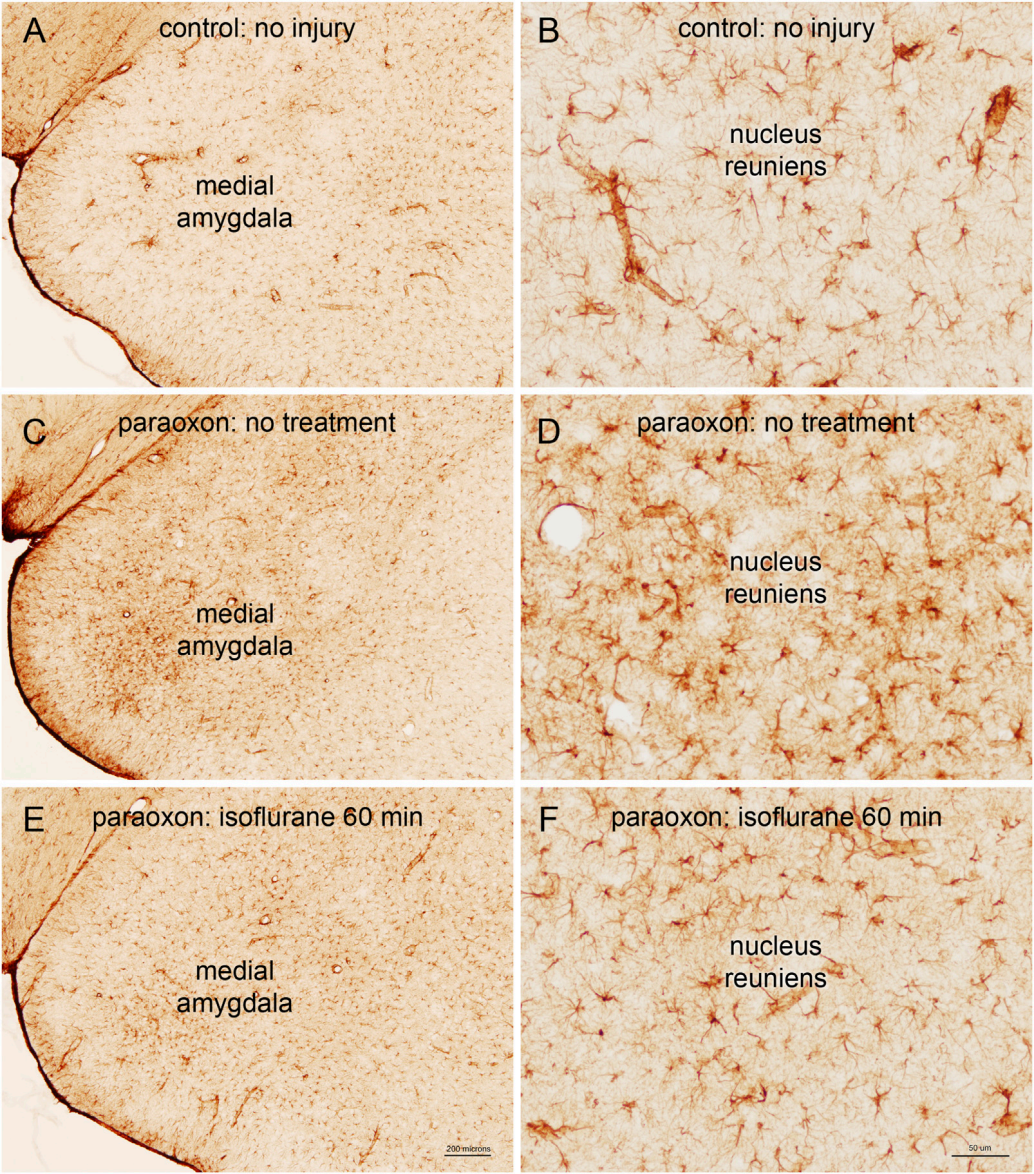
Astrogliosis in response to paraoxon and paraoxon plus isoflurane. Glial fibrillary acid protein (GFAP) is a marker for astrocytes, and reactive astrogliosis. GFAP staining in the brains of control (uninjured) animals was moderate **(A)**, and astrocyte morphology was indicative of resting astrocytes **(B)**. Twenty-four hours after paraoxon poisoning, GFAP staining was substantially increased, for example in the medial amygdala **(C)**, and astrocytes displayed morphology consistent with astrogliosis [e.g., enlarged cell bodies and processes; **(D)**]. In animals treated with isoflurane 60 min after paraoxon administration, GFAP staining was substantially reduced compared with untreated animals [comparing **(C–E)**]. Also, astrocyte morphology in the isoflurane treated animals was more consistent with resting astrocytes, for example in the nucleus reuniens of the thalamus [comparing **(D–F)**]. Representative images from n = 3 in each group.

We also examined astrogliosis in response to paraoxon and the effect of isoflurane treatment. GFAP staining in the brains of control (uninjured) animals was moderate, and astrocyte morphology was indicative of resting astrocytes. Twenty-four hours after paraoxon exposure, GFAP staining was substantially increased in various parts of the brain and astrocytes displayed morphology consistent with astrogliosis (e.g., enlarged cell bodies and processes). In animals treated with isoflurane 60 min after paraoxon administration, GFAP staining was substantially reduced compared with untreated animals. Also, astrocyte morphology in the isoflurane treated animals was more consistent with resting astrocytes, indicating a substantial reduction in astrogliosis (Figure 9).

## 4 Discussion

OP poisoning is a significant world health problem, claiming tens of thousands of lives per year through intentional and unintentional pesticide exposure (Mew et al., 2017; Boedeker et al., 2020). OP-based chemical agents, including certain common pesticides and chemical warfare nerve agents, exert their toxic effects through cholinergic over-activation during the initial phase. However, the central pathologies shift to a non-cholinergic phase which can lead to prolonged convulsions and seizures, excitotoxicity, irreversible neuronal loss and permanent CNS damage. Prognoses for OP poisoning involving post-exposure treatments are generally poor due to the fact that current medical countermeasures fail to fully control the later phase of non-cholinergic excitotoxicity. Hence there is an unmet need to develop and characterize novel therapeutics that rapidly and effectively block OP-induced convulsions and seizures. One particular focus of these efforts is on anti-glutamatergic agents which can suppress the secondary excitotoxic cascades induced by organophosphates (Pulkrabkova et al., 2023), but to date no anti-glutamatergic agent has been approved for the treatment of convulsions in OP poisoning patients.

Control of OP-induced seizures is critical for neuroprotection (Shih et al., 2003). The potential use of anesthetics as neuroprotectants in OP poisoning has been indicated in several earlier studies, most notably against OP nerve agents. Simultaneous anesthesia using isoflurane plus nitrous oxide was shown to interrupt soman-induced seizures and to attenuate edema in selected brain areas in mice (Testylier et al., 2007). In a later study, protective effects of several anesthetics on sarin poisoning were investigated in domestic swine (Sawyer et al., 2012). In this study 2% isoflurane was delivered in 100% oxygen continuously for 6 h and resulted in profound protection against sarin poisoning. The investigators did not examine shorter duration isoflurane administration or higher doses. High dose midazolam (5 mg/kg) was found to be moderately effective at preventing neural damage and reducing mortality when administered up to 1 h after sarin (Chapman et al., 2015). A more recent study on the use of midazolam in conjunction with other anticonvulsant drugs found that the combination of midazolam and isoflurane was the most effective combination in protecting the CNS from long term damage in a paraoxon exposure model (Swissa et al., 2020). The authors concluded that isoflurane was the only agent with significant neuroprotective effects. It is important to stress that Swissa et al. administered 2% isoflurane for 1 h, which is substantially different than our protocol employing 4%–5% isoflurane with a very short-administration time. In previous studies we have found that lower doses of isoflurane are not as effective at blocking convulsions and preventing neuronal loss (Krishnan et al., 2017). Other studies have also shown that low doses of isoflurane (1.2%–1.5%) are not effective in reducing edema in a cerebral hemorrhage model (Esposito et al., 2013). These findings indicate that the effect of isoflurane is concentration dependent and that higher doses of isoflurane provide greater neuroprotection. In the current study, we found that 4% isoflurane, delivered for 5 min, was as effective as 5% isoflurane in permanently blocking convulsions (Figure 1).

*In vivo* MRI findings in the current study demonstrate the robust neuroprotective effects of brief-duration, high-dose isoflurane, even when administered 1 h after a lethal dose of paraoxon. T2W images revealed significant brain edema in the group given paraoxon without isoflurane treatment (Figure 2). In contrast, the group treated with isoflurane at 1 h showed T2 values that were indistinguishable from the uninjured control group (Figure 2 inset). These findings indicate that isoflurane administration mitigates the development of brain edema following paraoxon-induced injury. Mean diffusivity reflects the freedom of water diffusion in brain tissue and can indicate tissue damage. Our results demonstrated a substantial reduction in MD in brain regions such as the neocortex in the paraoxon group, with a return to near control levels in the group treated with 5% isoflurane for 5 min at 1-h after paraoxon administration (Figure 3 inset). The decrease in mean diffusivity in the isoflurane-treated group suggests attenuation of cellular injury and preservation of tissue integrity. Increased T2 values observed on MRI and decreased MD values suggest the presence of cytotoxic edema in the brain in response to paraoxon poisoning. Cytotoxic edema refers to the swelling of brain cells (neurons and glial cells) due to intracellular accumulation of ions and water. It is often associated with cellular injury or dysfunction, such as ischemia, metabolic disturbances and toxic insults. On T2W MRI images, increased signal intensity (quantitatively increased T2 values) indicates an accumulation of fluid, while decreased mean diffusivity suggests restricted movement of water molecules within the brain tissue. This restriction occurs due to cellular swelling, which hinders the free diffusion of water molecules (Ho et al., 2012; Keep et al., 2017; Obenaus and Badaut, 2022). These T2 and MD changes derived from *in vivo* MRI provide evidence of the neuroprotective effects of brief-duration, high-dose isoflurane administered in response to paraoxon-induced injury. Isoflurane treatment effectively reduces brain edema, as observed in the T2-weighted images, and preserves tissue integrity, as indicated by the normalized mean diffusivity values. These findings support the potential therapeutic use of isoflurane in mitigating cytotoxic edema and promoting neuroprotection in OP poisoning.

Using FJB staining to show degenerating neurons, we found that isoflurane was very effective at preventing neuronal loss when administered 1 h after paraoxon. Less neuroprotection was observed when isoflurane administration was delayed until 90 min, and the neuroprotective effect was lost when isoflurane administration was delayed until 3 h after paraoxon (Figures 4–8). We also examined astrogliosis in response to paraoxon and treatment with isoflurane using GFAP immunohistochemistry. Twenty-four hours after paraoxon administration, astrogliosis was observed throughout the brain, especially in regions that also showed extensive neuronal loss such as the nucleus reuniens, neocortex and the amygdala. In animals treated with 5% isoflurane for 5 min, the astrogliosis was substantially reduced demonstrating reduced pathology (Figure 9).

The repurposing of isoflurane as an anticonvulsant drug indicated in OP poisoning appears warranted based on our findings. Our approach repurposes high-dose isoflurane as a single-dose neuroprotective drug, in contrast to its use as a surgical anesthetic with prolonged administration times using lower dosages. Isoflurane’s effectiveness in blocking OP-induced convulsions and reducing CNS damage is likely due to its actions on multiple distinct receptor and channel systems (Nishikawa and MacIver, 2000; Sun et al., 2015; Xiao et al., 2015; Li et al., 2016; Neag et al., 2020). For example, the strength of synaptic signaling in the amygdala mediated by glutamate receptors (NMDA and non-NMDA) and GABA_B_ receptors was decreased by isoflurane, whereas GABA_A_ receptor-mediated signaling was increased (Ranft et al., 2004). It has also been reported that isoflurane reduces ischemia-induced glutamate release (Ritz et al., 2006). In brain slice preparations, isoflurane strongly reduced synaptic transmission, network oscillations and calcium influx into neurons, and reduced cerebral metabolic rate (Berndt et al., 2021). Isoflurane also inhibits mitochondrial complex I, which reduces presynaptic ATP levels and inhibits synaptic vesicular endocytosis, thus reducing synaptic activity (Jung et al., 2022). The combined effects of these actions provide neuroprotection in both pre-conditioning and post-conditioning scenarios (Jiang et al., 2017).

The efficacy of isoflurane in OP poisoning may also be related to its actions on TASK-1 and TASK-3 potassium channels, which act to hyperpolarize neuronal membranes and reduce excitability. Isoflurane activates TASK-1/3 channels with subsequent neuroprotective effects (Liu et al., 2005; Yao et al., 2017). TASK-1/3 potassium channels are strongly expressed in neurons throughout the CNS (Talley et al., 2001; Fan et al., 2022) as well as in mitochondria (Szewczyk et al., 2009). Numerous studies have found isoflurane to have neuroprotective and cardioprotective properties in various injury models through actions on TASK-1/3 and other potassium channels (Liu et al., 2005; Park et al., 2005; Khatibi et al., 2011; Taheri et al., 2014; Altay et al., 2020; Zhai et al., 2023). Studies on TASK-1 and TASK-3 knockout mice suggest that TASK-3 channels are the potassium channels most involved in the anesthetic actions of halogenated ethers including halothane and isoflurane (Pang et al., 2009) and mutagenesis studies have provided support for this conclusion (Luethy et al., 2017). By targeting systems in addition to the GABA_A_ receptor, isoflurane can provide an effective adjunct anticonvulsant and neuroprotectant to the current medical countermeasures for OP poisoning.

However, what is not clear is how such brief exposures to isoflurane can permanently block convulsions, as isoflurane is expelled rapidly from the body through exhalation. In earlier studies, Bar-Klien and others found that isoflurane greatly reduced the incidence of seizures, prevented blood-brain barrier damage, reduced neuronal loss and prevented astrogliosis in a paraoxon model of injury (Bar-Klein et al., 2016). Our results are in excellent agreement with their findings, despite major differences in the methodologies used. In their paraoxon model, they used a much lower dose of paraoxon (0.45 mg/kg) that was approximately 9 times lower than the dose we employed (4 mg/kg). Further, Bar-Klein et al. used multiple 1-h administrations of 1%–2% isoflurane delivered in 100% oxygen, and this treatment was given at 1, 6 and 12 h, 1, 2, 3, 7 and 30 days after paraoxon administration. This use of isoflurane as an anesthetic is in sharp contrast to our delivery of 4%–5% isoflurane administered in 100% oxygen for 5 min in a single dose. Despite the different approaches, the results were very similar in the two studies. The novelty of our approach was the discovery that longer duration administrations and repeated applications of isoflurane were not necessary to achieve its potent anticonvulsant and neuroprotective effects.

Prolonged exposure to isoflurane has revealed cognitive deficits in animal models, and in infants and elderly patients following surgery with isoflurane anesthesia. In one animal study, 20-month-old rats were exposed to 1.3% isoflurane for 4 h and spatial learning and memory were assessed at 2 weeks after isoflurane exposure (Kong et al., 2015). Isoflurane induced learning and memory deficits under these conditions. In another study, mice were exposed to 0.75% isoflurane for 4 h three times at postnatal days 7, 8 and 9 and contextual fear conditioning was tested at 3 months after the anesthesia administration. These mice showed significant memory impairment. (Zhong et al., 2015). These and similar studies indicate that prolonged isoflurane exposure can have adverse effects especially in neonatal and elderly populations (Mandal et al., 2009; Deng et al., 2014; Coleman et al., 2017; Kang et al., 2017; Liu et al., 2017; Noguchi et al., 2017; Belrose and Noppens, 2019). Our treatment regimen is designed to maximize effectiveness and minimize any potential side effects by using very brief, 5-minute exposures to isoflurane. This approach will also limit the possibility of eliciting rare side effects of isoflurane, such as malignant hyperthermia, which can affect between 1:10,000 to 1:250,000 people (Smith et al., 2018). Malignant hypothermia onset is dependent on the duration of anesthesia and is more common in patients also receiving succinylcholine (Visoiu et al., 2014). Brief exposure times would not elicit this pathological response, even in sensitive patients.

Taken together, these results show that isoflurane is an effective anticonvulsant and neuroprotectant that can be used in conjunction with benzodiazepines to treat OP-exposed patients. In our studies isoflurane halted paraoxon-induced convulsions rapidly, and the convulsions did not return at any time point after cessation of brief isoflurane administration throughout an 8-h post-exposure observation period. Because OP-induced convulsions can become refractory to benzodiazepine treatment, any adjunct treatment that provides additional anticonvulsant action is desirable. Here, short-duration isoflurane administration at a concentration of 4%–5% has proven very effective in controlling convulsions and preventing neuronal loss when administered up to 1 hour after a lethal dose of paraoxon. Further, isoflurane provides more neuroprotection in OP-poisoning than benzodiazepines (Swissa et al., 2020). While our results are encouraging, studies that include larger experimental groups with EEG monitoring of seizure activity will be needed to provide more accurate measures of the time course of effectiveness of isoflurane in blocking seizures and reducing neuronal loss due to OP poisoning. Additional studies are ongoing with continuous EEG monitoring in soman animal models to determine if brief, high-dose isoflurane is as effective when used to treat OP poisoning involving more potent cholinesterase inhibitors.

## Data Availability

The raw data supporting the conclusion of this article will be made available by the authors, without undue reservation.
